# *RADX* Gene Variant May Predispose to Familial Asperger Syndrome

**DOI:** 10.3390/genes14020301

**Published:** 2023-01-23

**Authors:** Alessia Azzarà, Roberto Rumore, Fulvia Brugnoletti, Elisabetta Tabolacci, Irene Bottillo, Eugenio Sangiorgi, Fiorella Gurrieri

**Affiliations:** 1 Research Unit of Medical Genetics, Department of Medicine and Surgery, Università Campus Bio-Medico di Roma, 00128 Rome, Italy; 2 Istituto di Medicina Genomica, Fondazione Policlinico Universitario A. Gemelli IRCCS—Università Cattolica del Sacro Cuore, 00168 Roma, Italy; 3 Oncogenetics Unit, Service of Oncology, University Hospital of Geneva, 1205 Geneva, Switzerland; 4 Division of Medical Genetics, Department of Experimental Medicine, San Camillo-Forlanini Hospital, Sapienza University, 00152 Rome, Italy; 5 Operative Research Unit of Medical Genetics, Fondazione Policlinico Universitario Campus Bio-Medico, 00128 Rome, Italy

**Keywords:** Asperger syndrome, exome sequencing, autism spectrum disorders, candidate gene

## Abstract

Asperger syndrome (AS) is a pervasive developmental disorder characterized by general impairment in socialization, stereotypical behavior, defective adaptation to the social context usually without intellectual disability, and some high functioning areas related to memory and mathematics. Clinical criteria are not well defined and the etiology is heterogeneous and mostly unknown. Like in typical autism spectrum disorders (ASD), the genetic background plays a crucial role in AS, and often an almost mendelian segregation can be observed in some families. We performed a whole exome sequencing (WES) in three relatives of a family with vertical transmission of AS-ASD to identify variants in candidate genes segregating with the phenotype. Variant p.(Cys834Ser) in the *RADX* gene was the only one segregating among all the affected family members. This gene encodes a single-strand DNA binding factor, which mediates the recruitment of genome maintenance proteins to sites of replication stress. Replication stress and genome instability have been reported recently in neural progenitor cells derived from ASD patients, leading to a disruption of long neural genes involved in cell–cell adhesion and migration. We propose *RADX* as a new gene that when mutated could represent a predisposing factor to AS-ASD.

## 1. Introduction

Autism spectrum disorder (ASD) is a heterogeneous group of neurodevelopmental conditions characterized by defective social communication and interaction, difficult adaptation to external stimuli, stereotypical and repetitive behavior, along with intellectual disability [1]. Asperger syndrome (AS), described for the first time in 1944 [2], defines a subgroup of patients with ASD usually with normal intelligence and normal language development, with some specific areas, such as memory and/or mathematical reasoning and/or artistic skills, not only preserved, but also showing a functioning above the average population. Typical autistic symptoms could be less evident and could range from inappropriate social behavior, lack of empathy, eye contact, and sarcasm, to more invalidating stereotypical obsessive behavior. Sometimes, exceptional mnemonic, mathematical, and artistic skills are present, and the IQ score results are well above the average population, allowing individuals with AS to have a near-normal life. However, AS patients more often than the general population show psychiatric comorbidities such as major depressive disorder, attention deficit hyperactivity disorder, obsessive–compulsive disturb and anxiety [3].

In order to define the etiology of ASD and AS, during the last 10 years, whole genome sequencing, whole exome sequencing, SNP-array, and comparative genomic hybridization (CGH) have been used to identify, in several different cohorts of sporadic or familial ASD patients, a plethora of molecular defects [4,5,6]. DNA lesions encompass copy numbers variants (CNV) and single nucleotide defects in several different genes; however, the most recurring variants are represented by CNV in specific chromosomal regions [7,8]. For this reason, SNP-array or CGH are routinely tested in a diagnostic setting to try to identify the molecular defect present in a patient with ASD. A search of the OMIM database returned between 26 and 30 susceptibility loci, highlighting the extreme genetic heterogeneity of this condition. Those loci represent just those validated in more than one study or in more than one individual but, if we consider all the genes identified in single families, the number of loci involved is much larger. It is well known that the ratio between male to female is 4 to 1, and one potential explanation relies on the protection offered to females by the X chromosome. A few loci are mapped on the X chromosome [9,10]. These findings suggest that the genetic heterogeneity of ASD-AS is larger than anticipated and probably a more complex genetic model must be taken into account to explain how many different genes and pathways can lead to this complex phenotype. We investigated a large family where AS segregated in several individuals along with psychiatric problems. In order to identify one or more variants present in all affected individuals, we performed whole exome sequencing and we identified a c.2500T>A in the *RADX* gene leading to p.(Cys834Ser) protein substitution. This gene encodes a single-strand DNA binding/maintenance protein.

## 2. Materials and Methods

### 2.1. Clinical Assessment

**Figure 1:**
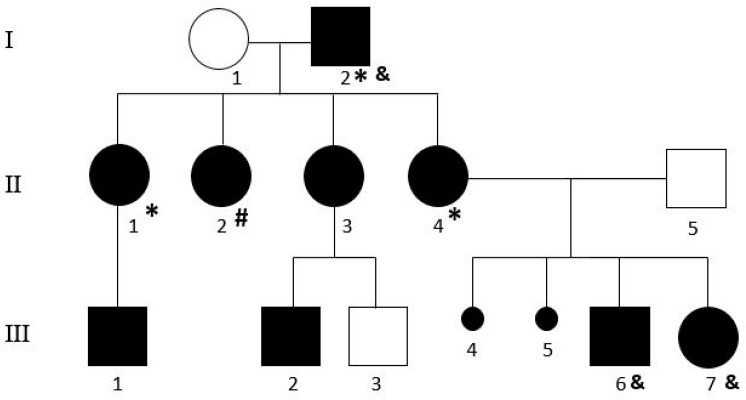
Pedigree of the family. “*” and “#” indicate the individuals carriers of arr[GRCH37]1q25.2(178443054_178964320)x3 and arr[GRCH37]Xp22.33(1217017_1378646)x3, respectively. “&” indicates the subjects in which the WES was performed.

We counselled a mother (II4) with her son and daughter (III6 and III7), formally diagnosed AS by a neuropsychiatrist: they requested and gave consent to investigate a possible genetic cause of their condition (Figure 1).

She was an aerospace engineer, and she received a prize for solving mathematical equations. She is currently unemployed, after working for several years for a major Italian space agency, due to her lack of adaptation to teamwork. To personally testify her uncommon skills, we provided her with the raw data from the exome analysis and, without any formal training in genetics or in next-generation sequencing, she was able to fully analyze the data, reaching the same conclusion as us but in shorter time. She was well aware of the condition of her two children: they both showed extreme shyness, anxiety, and behavioral issues. During our interview, she also reported that her father (I2), without any formal education or training in mathematics or mechanics, was able to solve complex equations and was awarded an honorary college degree for his achievement. He also built an engineering firm and he was a consultant for other companies to solve engineering construction problems. However, his skills were not matched by his social manners and his general behavior was described as rude and, in many cases, socially inappropriate. He had three other daughters and we could interview two of them: individual II1 got a college degree in astrophysics discussing a thesis on new mathematic equations. She maintained a balanced lifestyle as a high school teacher but suffered personal problems since she was very young and she has been in therapy every one/two years with a psychiatrist for the last 10 years. Her son is in psychotherapy as well for some unspecified social problems. The other daughters (II2 and II3) were also referred as socially impaired and one of them (II3) had a son with a diagnosis of AS (III2). DNA was extracted from a peripheral blood sample of each family member, using standard procedures, after signing the informed consent.

### 2.2. Exome Sequencing and Bioinformatics Analysis

WES analysis of I1 III6 and III7 was performed on service at Galseq SRL (Bresso, Milan, Italy) requesting an average coverage of 60× on an Illumina platform. For each patient a pair of fastq files was obtained. These were subsequently mapped and filtered using the online platform Galaxy [11] using a customized bioinformatics pipeline: we mapped paired reads using BWA [12], then removed duplicates and performed variant calling using FreeBayes. The ensuing vcf files from all three patients were merged using “bcf tools merge” to create a multisample vcf file. Then, we filtered again the whole file using regular expressions to identify variants shared by all three individuals. We filtered and obtained approximately 91,000 variants that were annotated using wAnnovar [13]. All bioinformatics analyses were carried out following best practice recommendations [14]. To evaluate the frequency of each variant, we searched the gnomAD database, while the in-silico analysis of each missense variants was performed using the website Varsome.com. The variant annotated file was then filtered using functions on Microsoft Excel. All the variants were then prioritized using Varelect [15] with the list of genes produced from our analysis and the following keywords (“brain”, ”autism”, ”Asperger syndrome”, ”neurons”, ”brain development”). From each search, a list of genes was generated. All the genes and their variants were manually inspected loading all the reads as a custom track on the UCSC Genome Browser to confirm their presence.

### 2.3. Sanger Sequencing

To perform segregation analysis, oligonucleotide primers flanking variants were designed using the Primer3 application on the UCSC genome browser. Primer sequences will be provided upon request. Each amplicon was PCR amplified and were purified with a 1:1 mixture of Exonuclease III and shrimp alkaline phosphatase at 37 °C for 15 min followed by heat inactivation at 80 °C. After cleaning up, the PCR product was sequenced using BigDye terminator v3.1 Cycle Sequencing Kit and run on a 3130 Genetic Analyzer (Applied Biosystems, Foster City, CA, USA). The electropherograms were analyzed by the Sequencing Analysis v5.2 software (Applied Biosystems, Foster City, CA, USA).

## 3. Results

In this family, several individuals presented a series of phenotypes ranging from a classical AS to social impairment, and/or anxiety and/or psychiatric-related illnesses along with exceptional mathematical and artistic skills. We considered affected all individuals with a formal diagnosis of AS as well as behavioral problems (Table 1).

As a part of the diagnostic workout, a CGH analysis was performed and detected a duplication on chromosome 1 (arr[GRCH37]1q25.2(178443054_178964320)x3) on I2, II2, and II4, while II2 had a duplication on the X chromosome (arr[GRCH37]Xp22.33(1217017_1378646)x3), probably inherited from her mother, not available for testing. Those CNVs were small, without a significant gene content and not segregated with the AS phenotype, since they were also absent in III6 and III7.

Since CGH was negative for pathogenic CNV, a WES analysis was performed on I2, III6, and III7, all of them with a formal diagnosis of AS, in order to attempt to identify the variant responsible for this phenotype. From the WES, approximately 91,000 variants were shared by the three affected individuals and were then included in the subsequent filtering process. In the next step, common variants (minor allele frequency > 1/1000) were then removed, along with synonymous, low-quality, and low-coverage variants located in non-coding regions. The remaining variants were prioritized on the Varelect website using different keywords to select genes involved in “brain”, “brain development”, “autism”, and “neurons”, and those variants were manually evaluated on the BAM file loaded as a custom track on the UCSC Genome Browser. The output of this search provided 18 variants in 18 different candidate genes that were followed up for segregation in the remaining individuals of the family (Table 2).

Among all those variants, only the c.2500T>A variant predicting a p.(Cys834Ser) in the *RADX* gene segregated in all affected individuals. In order to rule out variants missed during the prioritization procedure, we searched whether, among the shared 91000 variants, any were located in a known ASD or AS gene. From this search, no significant variant in this family was present in such genes. Since the X chromosome contains several loci previously involved in ASD-AS, we also genotyped our family with a panel of microsatellites mapping along the X chromosome. As expected, individual I2 passed his X chromosome to all his daughters and from this analysis the chromosomal region in linkage was approximately 100 Mb large going from nucleotide 3,891,000 to nucleotide 140,810,000 (data not shown). We searched in the literature all the genes/loci implicated in ASD-AS on the X chromosome that were located in our critical non-recombinant region, and we found that *NLGN3* was present in that region. We re-evaluated all the variants located in this region and manually inspected all the exons of *NLGN3,* and we ruled out the presence of other potentially pathogenic variants. In conclusion, we had only a segregating variant, the c.2500T>A p.(Cys834Ser) in the *RADX* gene: this variant is rare (1/182719 in the gnomAD database) and, according to Varsome database, has a negative impact on the function of the protein with most of the predictive software suggesting a damaging effect (Table 3). The CADD score for this variant is 25.1. On the consensus dataset, combining data from the HPA and GTEx transcriptomics database, the first five tissues where RADX had the highest expression were located in brain regions.

## 4. Discussion

In this work, we performed a WES in three relatives of a family with vertical transmission of AS-ASD to identify variants in candidate genes segregating with the phenotype. Segregation analysis showed that the variant in the *RADX* gene was present in all affected individuals, while it was absent in unaffected relatives. *RADX*, formerly known as *CXORF57*, encodes for an RPA1-related single-strand DNA binding protein. Its role was partially elucidated a few years ago during a bioinformatics screening of proteins containing an OB domain, showing that this protein is able to bind single-strand DNA [16]. The main factor binding single-strand DNA is RPA1, with its OB domains, that prevents, upon coating single-strand DNA at regions of replication stress, catastrophic collapse of a stalled replication fork. It also recruits proteins involved in DNA replication and DNA damage response [17]. RADX collaborates with RPA1 to establish replication integrity under basal and stressful conditions. In addition, RADX negatively regulates RAD51 accumulation at replication forks and this regulation prevents inappropriate RAD51-dependent fork reversal [18]. The proposed model indicates that RADX is an RAD51 antagonist that ensures the right amount of RAD51 fork reversal and protection activities to maintain genome stability. The variant found in our AS family is located outside the OB domains, although is predicted to have a damaging effect on the function of the protein by using common in silico predictive tools. In a recent paper, neural progenitor cells (NPC) derived from an ASD patient, display features of replication stress because of accelerated replication through the S-phase. Stalled replication fork leads to DSBs in recurrent hot spots, disrupting long genes involved in basic functions of NPC. Transcriptional analysis of those genes demonstrated a clear down-regulated expression in genes that were previously implicated in the pathogenesis of autism. Therefore, failure to cope with replication stress seems to be a new mechanism related to ASD. We hypothesize that, in this family, the variant in RADX could create a stalled replication fork and replication stress leading to recurrent double-strand breaks (DSBs) in the same genes previously reported [19]. This variant is predicted as damaging and this gene has a high expression in the brain. It is interesting that germline variants in *RAD51*, another gene involved in single-strand DNA maintenance, are responsible for a neurological condition, the congenital mirror movement syndrome, suggesting a role in neuronal function for this gene [20].

ASDs are significantly heterogeneous in their etiology and clinical manifestation. Many genetic and probably non-genetic (i.e., environmental) factors contribute to the complexity of ASD; since our study relates to one single family, these findings need to be confirmed in a larger cohort of individuals, and functional studies will help to confirm the role of this variant and of this gene in the pathogenesis of autism. These results might represent a significant step forward in the field of neurodevelopmental defects as they may contribute to confirm not only a new gene but also a new mechanism for ASD-AS and a new abnormal pathway to rescue with precision therapies.

## Figures and Tables

**Table 1:** Clinical description of the individuals of this family. We differentiated a formal AS diagnosis from a clinical diagnosis of a major psychiatric disorder. The “high-functioning side” of the AS syndrome was indicated as AS “features”.

Individual	I2	II1	III1	II2	II3	III2	II4	III6	III7
Formal diagnosis						AS	AS	AS	AS
Clinical diagnosis	Behavioral problems	Major psychiatric disorder	Major psychiatric disorder	Major psychiatric disorder	Major psychiatric disorder				
AS “features”	Mathematical/ engineering skills	Astrophysicist		Drawing skills			Aerospace engineer	Computer skills	

**Table 2:** Segregation analysis of 18 variants obtained from the filtering and prioritizing process.

Position ^†^	RefSeq Gene and HGVS Nomenclature ^‡^	II1	III1	II2	II3	II4	III2
chr1:150444527	*RPRD2*:NM_015203:exon11:c.3103C>T:p.(P1035S)	√	√	X			
chr12:2074822	*DCP1B*:NM_152640:exon5:c.424A>G:p.(T142A)	X		X	X		
chr12:95528593	*FGD6*:NM_018351:exon8:c. 3004 C>T:p.(R1002C)	√	X	X	X		
chr16:2263822	*PGP*:NM_001042371:exon2:c.873T>G:p.(N291K)	X		X	√		
chr16:84212975	*TAF1C*:NM_005679:exon14:c.2182C>G:p.(R728G)	√	√	X	X		
chr19:42819206	*TMEM145*:NM_173633:exon6:c.482G>A:p.(R161Q)	X		X	X	√	
chr19:49129547	*SPHK2*:NM_001204160:exon2:c.331C>A:p.(R111S)			X			X
chr2:15564564	*NBAS*:NM_015909:exon23:c.2452G>C:p.(E818Q)	X		√	√		
chr2:241569440	*GPR35*:NM_001195382:exon6:c.164A>T:p.(Y55F)	√	X	√	√	√	√
chr2:24522996	*ITSN2*:NM_001348182:exon12:c.1126A>G:p.(M376V)	X		√			X
chr2:71209151	*ANKRD53*:NM_001115116:exon4:c.703G>A:p.(A235T)	X			√		√
chr2:85828148	*TMEM150A*:NM_001031738:exon4:c.196A>G:p.(I66V)			X			
chr20:62164999	*PTK6*:NM_005975:exon4:c.575C>T:p.(T192M)	√	X	X			
chr4:83839213	*THAP9*:NM_024672:exon5:c.1848A>G:p.(L616L)	√	X	X			
chr5:180660683	*TRIM41*:NM_033549:exon5:c.1211C>T:p.(P404L)	√	√	X			√
chr6:44221229	*HSP90AB1*:NM_001271969:exon12:c.2069T>C:p.(I690T)	X					X
chr9:1056825	*DMRT2*:NM_181872:exon4:c.1238C>T:p.(T413M)	√	X	√	√	√	X
chrX:105921420	*RADX*:NM_001184782:exon13:c.2209T>A:p.(C737S)	√	√	√	√	√	√

**^†^** Human GRCh37/hg19. **^‡^** HGVS: Human Genome Variant Sequence. “√” indicates presence of the variant; “X” indicates the absence of the variant. In this analysis, the three individuals we analyzed by exome sequencing were not included.

**Table 3:** In silico evaluation of the *RADX* variant identified.

**Pathogenicity Scores**			
MetaLR	*prediction*	*score*	*rankscore*
	Damaging	0.5693	0.844
MetaSVM	*prediction*	*score*	*rankscore*
	Tolerated	−0.2734	0.7572
MetaRNN	*prediction*	*score*	*rankscore*
	Damaging	0.9322	0.9255
REVEL	*prediction*	*score*	*rankscore*
	Uncertain	0.578	0.8317
**Individual Predictions**			
BayesDel addAF	*addAF prediction*	*addAF score*	*addAF rankscore*
	Damaging	0.1129	0.6565
BayesDel noAF	*noAF prediction*	*noAF score*	*noAF rankscore*
	Tolerated	−0.0756	0.6523
FATHMM	*prediction*	*score*	*converted rankscore*
	Tolerated	−1.09, −1.35, −1.45	0.8089
FATHMM-MKL	*coding prediction*	*coding score*	*coding rankscore*
	Damaging	0.8163	0.4099
LIST-S2	*prediction*	*score*	*rankscore*
	Tolerated	0.7136, 0.7082, 0.7238	0.3374
LRT	*prediction*	*score*	*converted rankscore*
	Deleterious	0	0.8433
M-CAP	*prediction*	*score*	*rankscore*
	Damaging	0.1083	0.7848
MVP	*prediction*	*score*	*rankscore*
	Benign	0.3697	0.3658
MutPred	*prediction*	*score*	*rankscore*
	Pathogenic	0.811	0.9272
MutationTaster	*Prediction*	*Accuracy*	*converted rankscore*
	Polymorphism, Disease causing	0.89, 0.9971	0.3593
PROVEAN	*prediction*	*score*	*converted rankscore*
	Damaging	−4.95, −5.68, −5.47	0.8722
SIFT	*prediction*	*score*	*converted rankscore*
	Damaging	0	0.9125
SIFT4G	*prediction*	*score*	*converted rankscore*
	Damaging	0	0.9282

## Data Availability

Research data not shared.
